# Evaluating the cross-cultural competence instrument for healthcare professionals (CCCHP) among nurses in Okinawa, Japan

**DOI:** 10.1186/s12913-024-10814-6

**Published:** 2024-03-23

**Authors:** Chieko Shirai, Daisuke Nonaka, Jun Kobayashi

**Affiliations:** 1 Graduate School of Health Sciences, University of the Ryukyus, 207 Uehara, Nishihira-Cho, Nakagami-Gun, Okinawa, 903-0215 Japan; 2 Nakagami Hospital, 610 Noborikawa, Nakagami-Gun, Okinawa, 904-2142 Japan

**Keywords:** Cross-cultural competence, Japanese nurses, Psychometric properties, Scale validity and reliability, Multicultural care

## Abstract

**Background:**

To provide better quality healthcare services to patients with different linguistic and cultural backgrounds, the cross-cultural competence of medical professionals is important. However, assessing and improving the cross-cultural competence of healthcare professionals is difficult in Japan, as there is no standardized scale to measure the competence. This study’s purpose was to translate the Cross-Cultural Competence instrument for Healthcare Professionals (CCCHP), which was developed and used in Europe, and to examine its reliability and validity among Japanese nurses.

**Methods:**

During June and July 2021, nursing staff were invited to take web- and paper-based surveys in Okinawa Japan. The CCCHP (five-factor model with 27 items across motivation, attitude, skills, emotion, and knowledge) was translated using a combination translation method, and a five-point Likert scale was used for responses. Exploratory and confirmatory factor analyses and known-group method were used to examine structural validity, while Cronbach’s alpha coefficient was used to test reliability.

**Results:**

A total of 294 responses were analyzed; 77.2% had more than five years of experience. Since the fit index indicated that the five-factor model was not a good fit, it was modified to a four-factor model (J-CCCHP24) by moving three variables, removing the knowledge factor, and using the error covariance of the variables. The fit index after the modification was improved to comparative fit index (CFI) = 0.92, Tucker-Lewis index (TLI) = 0.91, root mean square error of approximation (RMSEA) = 0.05, and standardized root mean square residual (SRMR) = 0.06, and Cronbach’s alpha was 0.85. The mean scores of J-CCCHP24 were significantly higher in the group with a history of overseas travel, higher foreign language skill, training in intercultural care, experience of foreign patient care, and intercultural interactions outside the workplace than in the group without these characteristics.

**Conclusion:**

This study confirmed the validity and reliability of the modified Japanese version of the CCCHP (four-factor model with 24 items). The results suggest that the exposure to different cultures on a personal level may help improve nurses' cross-cultural competence. Further refinement of this scale for practical use would encourage the implementation of necessary countermeasures to improve the cross-cultural competence of Japanese healthcare professionals.

**Supplementary Information:**

The online version contains supplementary material available at 10.1186/s12913-024-10814-6.

## Background

Foreign patients in Japan have experienced several cultural barriers: when they use Japanese medical services they feel difficulties, such as a lack of attention to their culture, not being able to exercise their right of withdrawal, being approached based on their appearance, and a lack of awareness of implicit understanding [1]. It has been pointed out that having a low level of cross-cultural competence among healthcare professionals can lead to an exclusionary manner toward foreign patients and an assumption that the Japanese style of care is always correct [1, 2]. In order to provide better quality healthcare services to patients with different linguistic and cultural backgrounds, the cross-cultural competence of healthcare professionals is important [2–4]. It will help the professionals be aware of the differences in patients' cultural values, beliefs, habits and behaviors, and devise ways to effectively support them to best promote their health and wellbeing [2–5].

The government has promoted the inclusion of foreign residents through the universal health insurance plan since 2012, hence any medical facility should serve patients from different cultural backgrounds. However, hospitals and professionals are rather reluctant to accept foreign patients because of the difficulty in providing translator service and culturally diverse care, a lack of manpower due to the greater numbers of Japanese patients, and other reasons [6]. There are more than 8000 active hospitals in Japan [7], but only 73 medical institutions had been accredited for caring for foreign patients by the Japan Medical Service Accreditation for International Patients (JMIP) as of 2022 since its establishment in 2013. Indeed, medical institutions without JMIP accreditation continue to be the only option for many foreign patients. In addition to language, the prejudice held by healthcare workers that the care of foreign patients is somewhat more difficult, and the low level of cross-cultural competence to bridge the expectation gaps, limit the acceptance of foreigners by Japanese medical services [1].

### Cross-cultural competence self-assessment scale

A psychometric properties scale can help to provide a better understanding of weaknesses and suggestions to improve the cross-cultural competence among healthcare professionals [8–12]. It is desirable that the scale can also measure the knowledge and skills of transcultural care that are required in clinical settings for the multidisciplinary healthcare team.

In one study conducted in Japan, Sugiura compared the competence of former overseas nursing volunteers and nurses in public hospitals using a unique scale [9]. The scale consisted of 46 items in a five-factor model, with the factors being culture-specific knowledge, skills, general knowledge, a tendency to approach or avoid, and awareness of one’s own culture. The reliability of the scale was confirmed by internal consistency and reproducibility, and the construct validity was ensured by factor analysis. The problem pointed out by Sugiura was the large number of items. In addition, six of the 11 observables in the skills category included some variables with missing values of 20% or more, possibly because the questions were not relevant and were difficult for respondents to answer.

In another study, Noji et al. translated the Caffrey Cultural Competence in Healthcare Scale (CCCHS), which was developed in the United States, to measure the cross-cultural competence of Japanese nursing staff [10]. The CCCHS is a shorter survey of 28-items with five factors: knowledge, transcultural nursing care within the work team, transcultural nursing care outside the work team, awareness of the limitations, and understanding of policy. Caffrey et al. longitudinally compared the CCCHS scores of two groups of nursing students between those receiving or not receiving nursing training in a foreign country [11]. In contrast, Noji et al. cross-sectionally measured the cross-cultural competence of nurses working in hospitals. The internal consistency was acceptable, but the five-factor model was not a good fit to Noji’s data without modification using error covariance. Neither the Japanese version of CCCHS nor the Sugiura scale has been utilized in clinical practice. One reason for this could be the lack of versatility in applying it to multidisciplinary healthcare teams other than nursing staff.

This study adopted the Cross-Cultural Competence for Healthcare Professionals (CCCHP) instrument developed in Germany. It can be used regardless of the healthcare specialty, verified with data from medical students and clinical psychologists [8]. The original CCCHP was in German, and the English version was created by those of the authors who are fluent in both German and English through the forward translation process. The 27-item model measures healthcare workers’ cross-cultural competence and factors through five factors: motivation and curiosity (MC), attitude (A), knowledge and awareness (KA), emotion and empathy (EE), and skill (S). A higher score indicated higher cross-cultural competence calculated on a five-point Likert scale. Questions are asked about daily clinical practice; for example, S 50 = “For patients who do not fully understand Japanese, I take more time than usual to explain treatment options to them.” In addition, the CCCHP comes with a sixth factor, social desirability (five items), which measures respondents’ tendency to choose a socially desirable response (a confounding factor) to ensure that intercultural competence is not overestimated. The response time for the CCCHP is approximately 10–15 min, so it should be acceptable for use among busy healthcare professionals. The Cronbach’s alpha coefficient was as high as 0.87, ensuring internal consistency.

The CCCHP was later translated into Finnish by Hietapakka et al. and used to measure the cross-cultural competence of nursing staff [12]. The five-factor model was not a good fit for the Finnish data. Even though the knowledge and awareness (KA) factor is generally considered an essential part of cross-cultural competence [4, 5, 8], KA was removed to improve the goodness-of-fit indices, as well as one variable from the motivation and curiosity factor (MC58). As of yet, the CCCHP has not been studied in Asia or Japan. Japan has a unique monoethnic culture because of its geographic character and history, so the results may differ from those of the German and Finnish versions. Therefore, the five-factor model with 27 items should be tested.

The purpose of this study was to develop a Japanese version of a self-assessment tool of cross-cultural competence for healthcare professionals based on Bernhard et al.’s CCCHP and to evaluate its psychometric properties for factor validity and internal reliability among nurses in Okinawa, Japan.

## Methods

### Study subject

This cross-sectional study was conducted from June to July 2021 in Okinawa, Japan, where the authors are located. Since the CCCHP has 27 items plus social desirability (five items), the required sample size was estimated *n* = 320 using an *N*:q ratio (sample size/number of items) = 10:1 [13]. Okinawa prefecture has posted online a list of medical care facilities available for foreign languages in Okinawa [14]. The first author contacted these medical care facilities, and then out of a total of 16 facilities, 11 joined our study. Additionally, the first author contacted clinics, educational institutions, and public health centers to determine if they had treated any foreign patients in the past two years and if they were willing to cooperate in this study. In total, eight hospitals, eight clinics, three educational institutions, and some nurses indicated their willingness to do so. A total of 351 copies of the explanatory document were distributed by those representatives (e.g., nursing directors, nurse managers, and clinic directors) to the study subjects with nursing background. In order to maintain generalizability of the results, no exclusion criteria were established.

### Preparation of the survey

After permission to use the CCCHP had been obtained from one of the authors, the English version of the CCCHP was translated into the ‘Japanese-CCCHP’ and included the social desirable factor (Supplementary file 1) in four stages to ensure the quality of the translation: (1) Forward translation by a Japanese researcher and the first author, who have both lived and worked as nurses abroad; (2) back translation by a professional business translator; (3) back translation review by a native speaker with a Master’s degree in education; and (4) minor final edits by the co-authors. While maintaining the equivalence of translation and the accuracy of the results of this study, some wording was adjusted to suit the Japanese context. For example, “immigrants” was translated to “foreigners staying in Japan” instead of the Japanese word for “immigrants.” When the Immigration Control Act was amended in 2009 and 2018, the Japanese government emphasized that it was not an “immigration policy” but a measure against foreign workers who stay for a certain period of time [15]. In turn, we followed the Japanese media in using the terms “foreigners staying in Japan” and “foreign patients”.

The J-CCCHP27 without the social desirable factor was numbered according to the description by Bernhard et al.: MC, representing motivation/curiosity, had nine items; A, attitudes (four items); S, skills (five items); EE, emotions/empathy (five items); and KA, knowledge/awareness (four items). There were 10 items that would be difficult to answer if the participant had little experience caring for foreign patients (one item in MC, four items in EE, three items in S, and two items in social desirable). For example, MC17 = “I enjoy talking about migrated people’s experiences here.” and EE 63 = “I get impatient when a patient doesn’t understand.”. Therefore, at the beginning of the survey, we added a supplementary note: “If you feel difficulty in answering due to lack of experience, please answer by assuming ‘if you were …’.”

Slight modifications were made to the answer options in the Japanese version (Supplementary file 2). All versions used a five-point Likert scale, but the reinforcing word ‘completely’ or ‘fully’ was removed in the Japanese version, since it has been pointed out that Japanese people prefer to choose intermediate responses, which may increase the skewness or kurtosis of the score distribution depending on the combinations of multiple responses [16]. The German version contains a ‘not able to answer’ option, but neither the Finnish or the Japanese versions do. We considered the ‘not able to answer’ option to be irrelevant for the Japanese version, because we accepted the hypothetical responses of the participants who have limited experience dealing with foreign patients. With regard to scoring, five points were assigned to “Agree” for normal items whereas one point was assigned to “Agree” for reversal items.

A further 19 descriptive questions were added, including demographic questions (e.g., gender, age group, and licenses), questions about current work (e.g., workplace, years of experience, and number of foreign patients handled in the past), and questions relevant to the known group method. Based on the results of previous studies, we hypothesized that the mean of the scores would differ depending on the presence or absence of the following characteristics: experience of being abroad, cross-cultural nursing care training, (subjective) foreign language skills, and intercultural interactions outside the workplace.

In order to motivate nurses in the selected facilities to participate in this study, a mixed mode method was adopted, allowing them to choose between a web- or paper-based survey. The QR code and URL address of the web-based questionnaire, created by QuestionPro.com, were included in the explanatory document. Since some facilities (three hospitals and two clinics) noted that it would not be necessary to send paper copies of the questionnaire, a total of 144 copies were sent to five hospitals and six clinics. When the participants chose the paper version, they could return the completed survey in a pre-addressed envelope, rather than the authors collecting the surveys individually to minimize the sense of obligation.

### Statistical analysis

Statistical software RStudio (1.4.1717) and EZR on R Commander (1.52) were used. First, to assess the sufficiency of the sample size, the Kaiser–Meyer–Olkin (KMO) measure of sampling adequacy was checked to confirm it was above 0.5 [17]. Then, the distribution of scores was analyzed comprehensively from the following points of view; 1) mean and median values approximately equal, 2) skewness and kurtosis values in the range of -1 to + 1, 3) Shapiro–Wilk test with *p* > 0.05, and 4) linear quantile–quantile (Q-Q) plots [18]. By considering all the information together, an overall decision was made about whether the distribution of scores is approximately normal. Since this study was a validation of an existing scale, we did not remove any variables based on the results of item analysis alone. In this regard, “there is no need to be overly sensitive to the statistical features (other than validity) of item scores, since the items are not used in isolation, but are summed and incorporated into the scale scores,” as stated by Yoshida et al. [19].

The reliability of the scale was rated as follows: Cronbach’s alpha coefficient of 0.80 or higher indicates high internal consistency and less than 0.50 indicates low internal consistency [20]. One reason for low internal consistency is that some items measure different characteristics. Therefore, if the alpha coefficient is less than 0.50, irrelevant variables can be removed to obtain measurement accuracy.

Confirmatory factor analysis (CFA) and exploratory factor analysis (EFA) were conducted to test the factor validity of the J-CCCHP27. CFA used the maximum likelihood (ML) method to assess the five-factor model, in which each observed variable was associated with only one factor. Goodness-of-fit indices were calculated: comparative fit index (CFI), Tucker-Lewis index (TLI), root mean square error of approximation (RMSEA), and standardized root mean squared residual (SRMR). The numerical cut-offs for evaluating goodness-of-fit were a CFI and TLI ≥ 0.95 (or ≥ 0.90), RMSEA ≤ 0.06, and SRMR ≤ 0.08 [21, 22]. A poor fit is when CFI and TLI < 0.90, RMSEA ≥ 0.10, and SRMR > 0.08 [21].

EFA checked one factor associated with at least three observables [8]. The number of factors was determined by parallel analysis and scree plots where an eigenvalue is greater than 1.0 in the parallel analysis together with the eigenvalue of the random data [23]. EFA was conducted by the principal factor method and promax rotation, which is recommended to use when the factors have been known to be correlated [23]. Promax rotation helps to achieve a simpler structure for interpreting the relation between factor and observed variables by making factor loadings more robust. After confirming that the commonality of each observed variable did not exceed 1.0, factor loadings of 0.40 or higher were sufficient to indicate an association with the factor [23].

### Model modification

In the case of poor fit, model modification should be considered to ensure the accuracy of the results calculated by the scale [22, 24]. Along with the logical reasons to modify, a modification index of 20 or more and the EFA results could statistically use a model with a better goodness-of-fit in this study. Although the continuity of the CCCHP study may be lost, the search for a model with a better fit for this study will be beneficial in finding a tool to measure cross-cultural competence among Japanese healthcare professionals. After the scale was modified, the distribution of the total score was checked using a quantile–quantile (Q–Q) plot. If the probability plots were aligned in a straight line, the distribution could be read as a normal distribution. In order to evaluate the effect of the modification, the Akaike information criterion (AIC) values before and after the modification were compared and smaller AIC indicated improvement [23].

### Known group method

The modified version of the J-CCCHP24, a four-factor model with 24 items with an improved goodness-of-fit and Cronbach’s alpha coefficient, was used with the known group method to evaluate convergent validity. Differences in the scores between groups were assessed using the Student’s *t*-test for two groups, one-way ANOVA for age groups, and Spearman’s correlation coefficient for ordered categorical groups. As hypothesized, a significant difference in scores (*p* < 0.05) between the groups meant that the modified J-CCCHP could measure the cross-cultural competence of the respondents.

### Ethical considerations

This study was conducted with the approval of the Ethical Review Committee for Medical Research Involving Human Subjects of the University of the Ryukyus (approval no. 1784). The distributed explanatory document described the main idea and purpose, assured anonymity, and explained that there was no disadvantage in participating. It also clearly stated that the submission of responses online or by mail would be considered as consent for participation.

## Results

A total of 294 complete responses were analyzed in this study (71.5% response rate). The web-based survey was well accepted by our participants, 222 responses were collected by the web-based survey and 72 responses by the paper-based survey. The KMO value for *n* = 294 was 0.87, which met the criteria for a sufficient sample size.

A descriptive summary of the participants in this study is shown in Table 1. The majority of participants were female (82.7%) and certified as registered nurses (72.5%) without upgraded certifications such as public health nurse or midwife. Only 1% were assistant nurses. Their main workplace was the inpatient ward (66.7%), and most of them had more than five years of nursing experience (77.2%). Only 4% had no experience caring for foreign patients.

**Table 1:** Characteristics of participants (*n* = 294)

		*n*	%
Gender	Female	243	82.7
	Male	51	17.3
Nationality	Japanese	293	99.7
	Non-Japanese	1	0.3
Age groups	20’s	71	24.1
	30’s	82	27.9
	40’s	86	29.3
	50’s and over	55	18.7
Certification	Nurse	213	72.5
	Assistant nurse	3	1.0
	Nurse with public health and/or midwife qualifications	78	26.5
Current workplace	Hospital inpatient	196	66.7
	Hospital outpatient	27	9.2
	Clinic outpatient	11	3.7
	Others	60	20.4
Work experience (cumulative)	0 to 5 years	67	22.8
	More than 5 years	227	77.2
Time abroad (cumulative)	None	82	27.9
	Less than 1 week	95	72.1
	1 week to 3 months	78	
	More than 3 months	39	
Number of foreign patients (cumulative)	None	11	3.7
	1 to 5	117	39.8
	6 to 15	89	30.3
	More than 15	77	26.2

The results of the item analysis are shown in Table 2. The total score of the J-CCCHP27 and subscales A and EE were normally distributed, but MC and S were not normally distributed with an upward bias toward high scores, and KA showed a sharp peak at the intermediate responses. For each item, KA 11 and EE 18 were normally distributed, KA 9 had a floor effect, and the other 24 items were non-normally distributed with either a rightward or ceiling effect.

**Table 2:** Item analysis

*n* = 294		Mean	SD	Median	Skewness Kurtosis (-1 < sk < + 1)	Shapiro–Wilk *p* > 0.05	Q-Q Plot	Overall normality judgement	Cronbach’s α
J-CCCHP27		102.54	11.14	101.5	Yes	No	Probably Yes	Yes	.83
J-CCCHP24		92.56	10.78	92	Yes	Yes	Probably Yes	Yes	.85
J-CCCHP22		81.78	9.43	81	Yes	Yes	Probably Yes	Yes	.85
MC	MC1	4.32	0.79	4	No	No		No	
	MC10	4.22	0.78	4	Yes	No		No	
	MC12	4.61	0.60	5	No	No		No	
	MC17	4.44	0.78	5	No	No		No	
	MC29	4.26	0.86	4	No	No		No	
	MC38	4.12	0.95	4	No	No		No	
	MC42	4.51	0.67	5	No	No		No	
	MC58	4.38	0.69	4	No	No		No	
	MC64	3.29	1.15	3	Yes	No		No	
	J-CCCHP27	38.14	4.91	39	No	No	No	No	.84
	J-CCCHP24	29.54	4.24	30	No	No	No	No	.84
	J-CCCHP22	33.76	4.60	34	No	No	No	No	.83
A	A8^a^	3.90	1.16	4	Yes	No		No	
	A21^a^	3.04	0.95	3	Yes	No		No	
	A43^a^	3.35	1.09	3	Yes	No		No	
	A60^a^	3.34	0.85	3	Yes	No		No	
	J-CCCHP27 & 22	13.63	2.73	13	Yes	No	Probably No	No	.59
	J-CCCHP24	16.76	3.29	17	Yes	No	Probably Yes	Yes	.62
KA	KA9^a^	1.85	1.06	2	No	No		No	
	KA11^a^	3.12	1.15	3	Yes	No		Yes	
	KA25	4.37	0.85	5	No	No		No	
	KA30^a^	3.76	1.30	4	Yes	No		No	
	J-CCCHP27	13.10	2.52	13	Yes	No	Probably No	No	.31
EE	EE18^a^	3.07	1.07	3	Yes	No		Yes	
	EE26^a^	2.74	1.32	2	No	No		No	
	EE48^a^	2.96	1.19	3	Yes	No		No	
	EE55^a^	3.52	1.17	3	Yes	No		No	
	EE63^a^	3.91	1.08	4	Yes	No		No	
	J-CCCHP27, 24 & 22	16.20	3.87	16	Yes	No	Probably Yes	Yes	.68
S	S5	4.29	0.70	4	Yes	No		No	
	S44	4.00	0.82	4	Yes	No		No	
	S50	4.23	0.74	4	Yes	No		No	
	S51	4.38	0.71	4	No	No		No	
	S53	4.57	0.63	5	No	No		No	
	J-CCCHP27 & 22	21.47	2.56	21	Yes	No	No	No	.75
	J-CCCHP24	30.07	3.44	30	No	No	No	No	.80

^a^reversal items

The Cronbach’s alpha coefficient for the entire scale was 0.83, which is satisfactory for high internal consistency, but for the subscales, only MC had a high value of 0.84, while the others ranged from 0.75 to 0.31. In particular, the Cronbach’s alpha coefficient of KA was the lowest at 0.31, indicating a too low internal consistency.

### Factor analysis

**Fig. 1:**
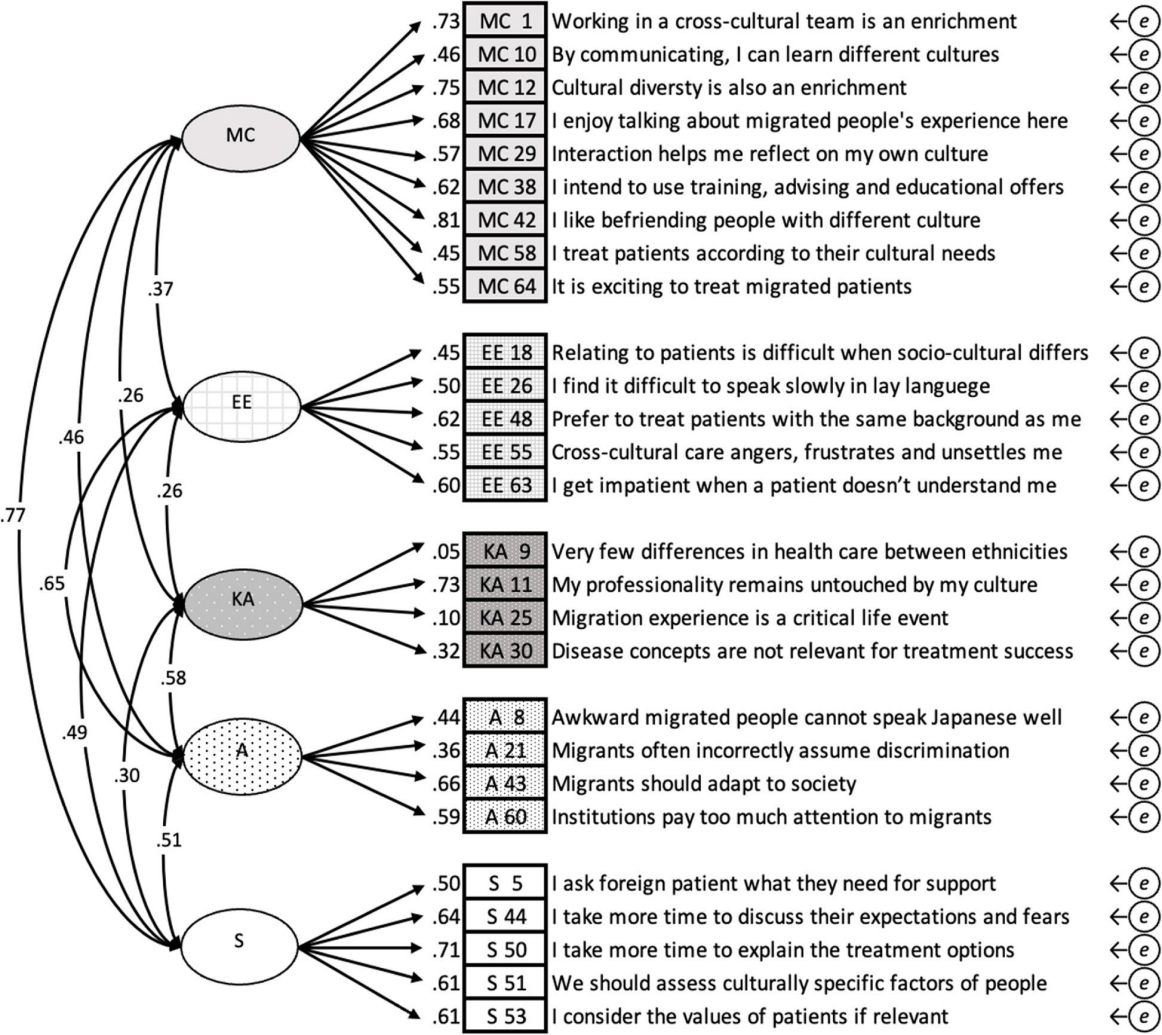
Confirmatory factor analysis of J-CCCHP27. The path diagram for the confirmatory factor analysis of the 27-item five-factor model. The goodness-of-fit indices were CFI = 0.81, TLI = 0.79, RMSEA = 0.07, SRMR = 0.08, AIC = 19318.8. Cronbach’s alpha coefficient is 0.83. (A: Attitude, EE: Emotion/Empathy, KA: Knowledge/Awareness, MC: Motivation/Curiosity, S: Skill, *e*: error covariance)

CFA indicated a poor fit for the five-factor model: CFI = 0.81, TLI = 0.79, RMSEA = 0.07, and SRMR = 0.08. Only SRMR met the borderline criteria (Fig. 1). Strong inter-factor correlations were found between MC and S, and A and EE. Several weak correlations below 0.30 were found between KA and the other factors also with observed variables. KA 9 and KA 25 were found to be uncorrelated (*p* > 0.05).

The EFA of J-CCCHP27 indicated four factors (Table 3), although the initial number suggested was five from the results of parallel analysis and scree plot. The factor loadings greater than 0.40 were extracted for MC, S, EE, and A, but the fifth constructional concept could not be extracted as a factor because there were only two observed variables with factor loadings above 0.40. In addition, KA 11 was rather associated with A instead of KA, and items MC 58 and 10 were associated with S instead of MC.

**Table 3:** Exploratory factor analysis of the J-CCCHP27

		EFA (principal factor method and promax rotation)						CFA (ML)	
		MC	S	EE	A	KA	Commonality	Correlation coefficient	Variance of error variables
MC	MC1	.84	-.12	-.09	.13	-.07	.58	.73	0.47
	MC10	.16	.34	.09	-.11	.19	.32	.46	0.79
	MC12	.59	.21	-.12	.09	.05	.56	.75	0.44
	MC17	.73	-.01	-.12	-.02	.04	.50	.68	0.53
	MC29	.61	-.02	.03	-.01	-.06	.35	.57	0.67
	MC38	.63	.00	.16	-.09	-.07	.44	.62	0.61
	MC42	.80	.02	-.11	.11	.01	.65	.81	0.35
	MC58	-.11	.81	-.12	.07	-.12	.50	.45	0.79
	MC64	.59	-.11	.44	-.14	-.12	.51	.55	0.69
A	A8^a^	-.01	.10	-.02	.44	.08	.23	.44	0.81
	A21^a^	-.12	.04	.05	.48	-.08	.25	.36	0.87
	A43^a^	.18	-.09	.14	.48	.10	.37	.66	0.56
	A60^a^	.19	.04	.03	.43	.12	.32	.59	0.65
KA	KA9^a^	-.13	-.30	.05	.01	.26	.16	.05	1.00
	KA11^a^	.07	-.07	-.14	.45	.43	.36	.73	0.47
	KA25	.19	.23	-.03	-.25	.25	.26	.10	0.99
	KA30^a^	-.06	-.05	.11	.04	.43	.19	.32	0.90
EE	EE18^a^	-.12	.01	.45	.05	.15	.24	.45	0.80
	EE26^a^	-.11	.02	.60	-.08	.18	.36	.50	0.75
	EE48^a^	.22	-.12	.62	.04	-.07	.46	.61	0.62
	EE55^a^	-.05	.04	.39	.29	-.11	.33	.55	0.69
	EE63^a^	.00	.13	.43	.12	-.02	.30	.60	0.64
S	S5	-.05	.47	.10	.13	-.04	.27	.50	0.75
	S44	.08	.45	.07	.07	.15	.38	.64	0.59
	S50	.14	.45	.08	.07	.11	.43	.71	0.50
	S51	.12	.65	-.10	.06	-.13	.49	.61	0.62
	S53	-.12	.76	.10	-.07	-.02	.49	.61	0.62
SS loadings		3.7	2.68	1.73	1.45	0.74			
Proportion var.		0.14	0.1	0.06	0.05	0.03			
Cumulative var.		0.14	0.24	0.3	0.35	0.38			

^a^reversal items

### Modification of the model

**Fig. 2:**
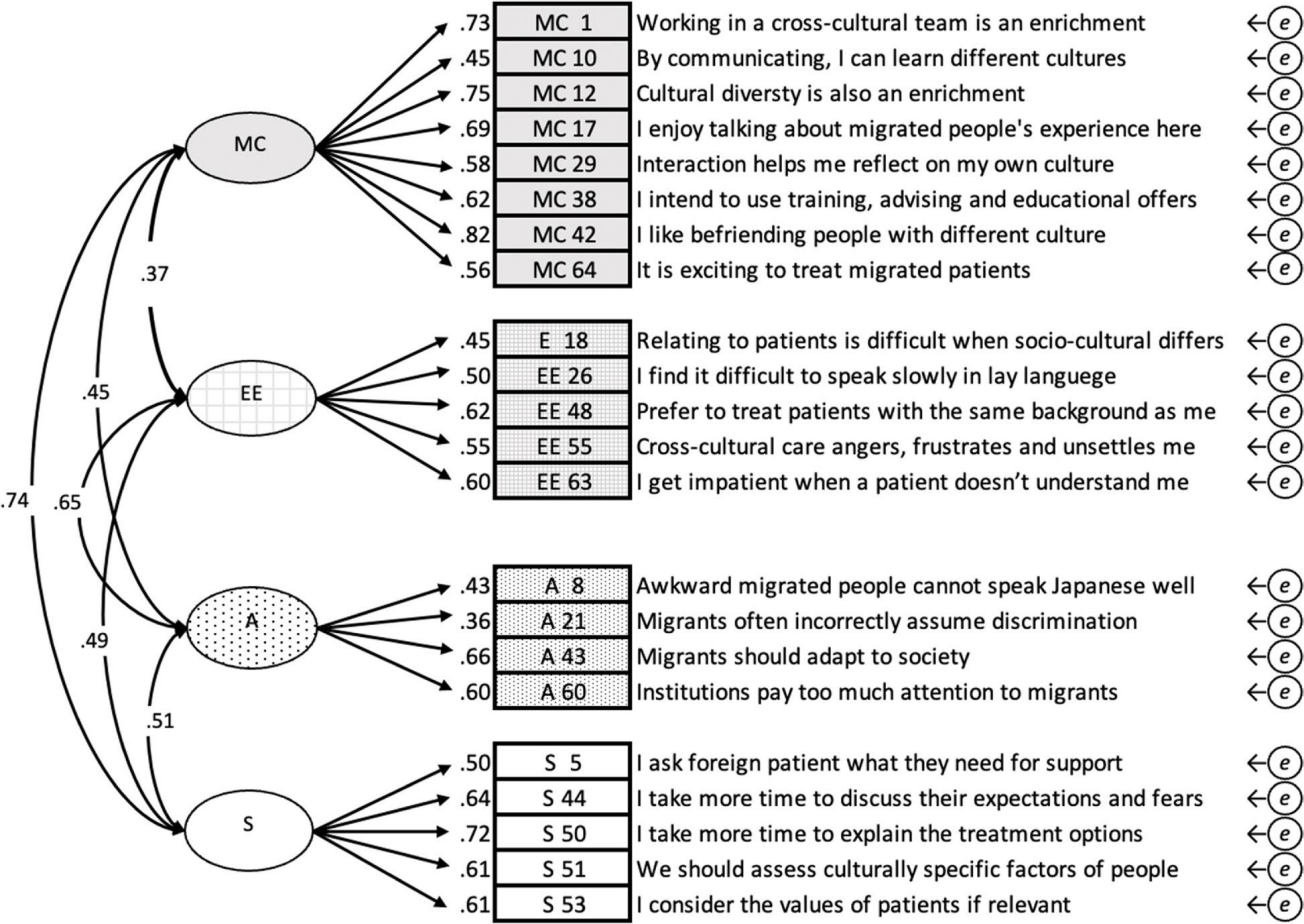
Confirmatory factor analysis of J-CCCHP22. The path diagram for confirmatory factor analysis of the 22-item four-factor model [J-CCCHP-22] maintained the modification of the Finch version. The goodness-of-fit indices were CFI = 0.87, TLI = 0.85, RMSEA = 0.06, SRMR = 0.06, AIC = 15279.7. Cronbach’s alpha coefficient is 0.85. (A: Attitude, EE: Emotion/Empathy, MC: Motivation/Curiosity, S: Skill, *e*: error covariance)

In order to achieve acceptable goodness-of-fit to ensure the reliability of the scores, model modification was required. The same modification as the Finnish version of the CCCHP was tried with four factors consisting of 22 items, named J-CCCHP22 (Fig. 2). In this model, variables (K 9, 11, 25, and 30 and M 58) were removed [12] and improved the goodness-of-fit significantly as CFI = 0.87, TLI = 0.85, RMSEA = 0.06, SRMR = 0.06, and AIC = 15,279.7. The total score of J-CCCHP22 was normally distributed and the Cronbach’s alpha was also improved to 0.85 (Table 2), however, the Cronbach’s alpha of subscale “A” was on the lower borderline of 0.59.

**Fig. 3:**
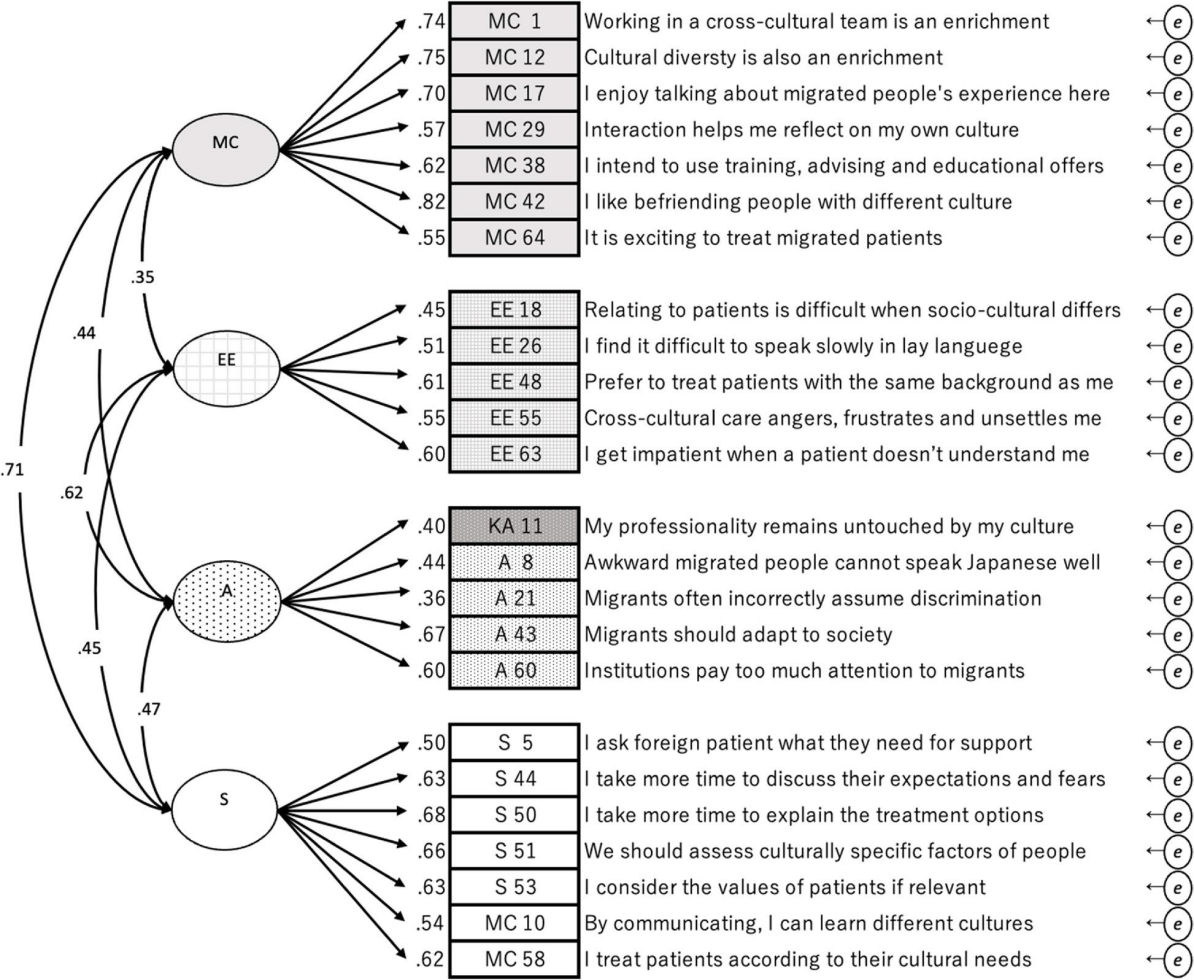
Confirmatory factor analysis of J-CCCHP24. The path diagram for confirmatory factor analysis of the 24-item four-factor model [J-CCCHP-24]. The goodness-of-fit indices were CFI = 0.88, TLI = 0.86, RMSEA = 0.06, SRMR = 0.06, AIC = 16654.5. Cronbach’s alpha coefficient was 0.85. (A: Attitude, EE: Emotion/Empathy, MC: Motivation/Curiosity, S: Skill, *e*: error covariance)

**Fig. 4:**
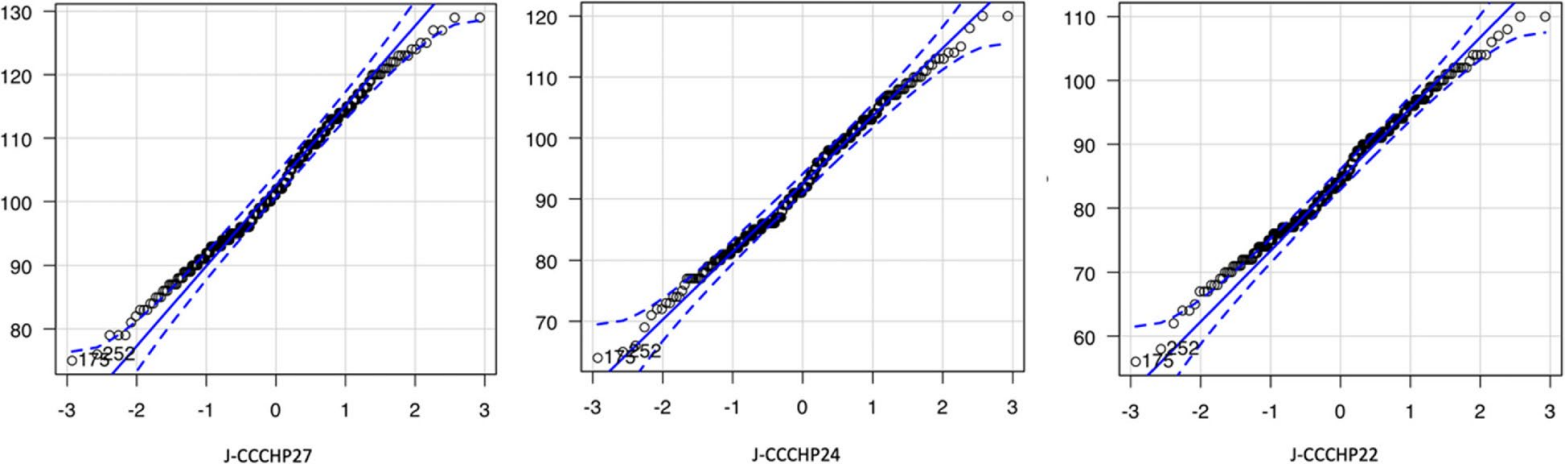
Q**-**Q plots. A comparison of Q-Q plots of J-CCCHP27 with 5 factor model, J-CCCHP24 with 4 factor model, and J-CCCHP22 with 4 factor model. The points lie mostly along the straight diagonal line with some minor deviations, but J-CCCHP24 had fewer outliers from the plot

**Fig. 5:**
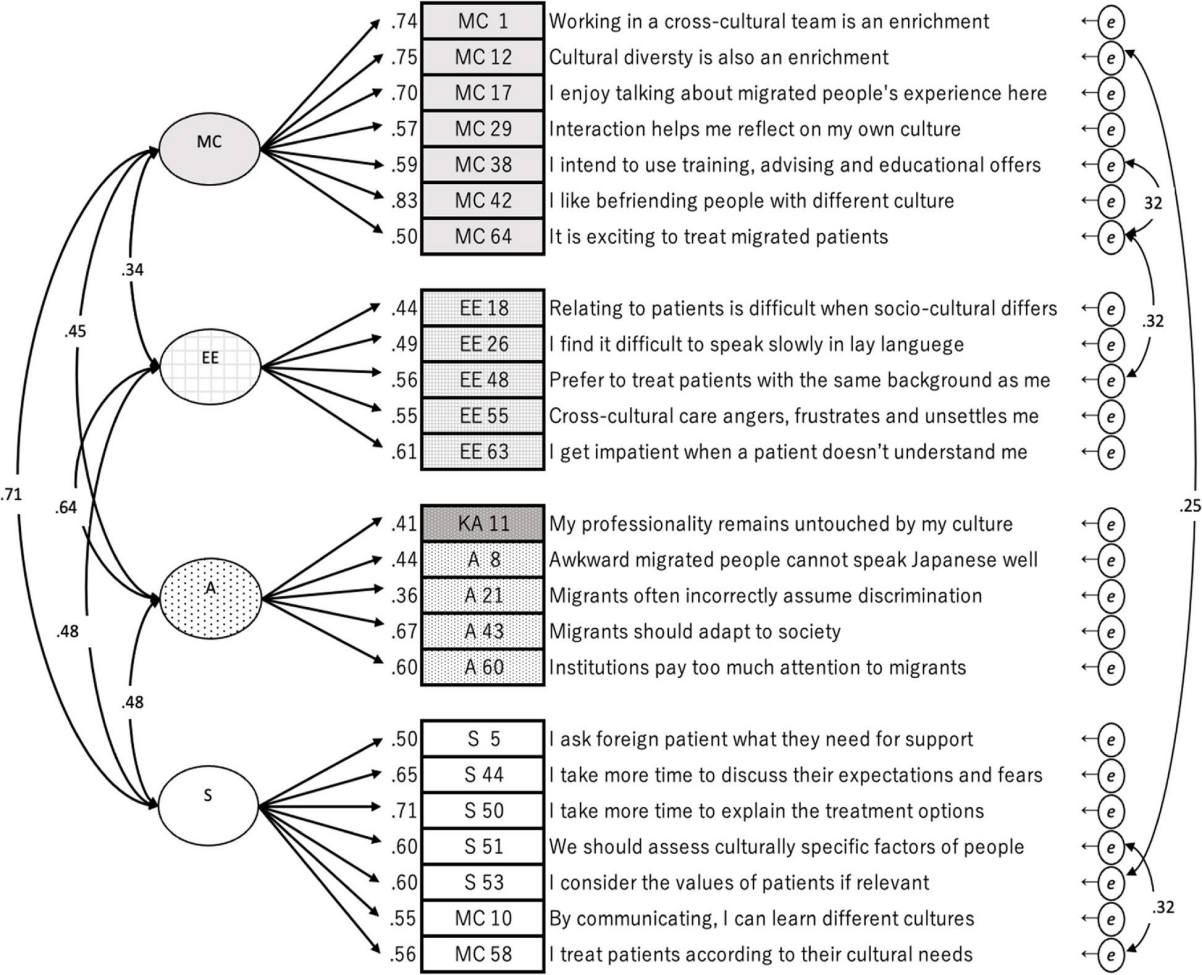
Confirmatory factor analysis of J-CCCHP24 with error covariances. The path diagram for confirmatory factor analysis of the 24-item four-factor model [J-CCCHP-24]. The goodness-of-fit indices were CFI = 0.92, TLI = 0.91, RMSEA = 0.05, SRMR = 0.06, AIC = 16559.6. Cronbach’s alpha coefficient was 0.85. (A: Attitude, EE: Emotion/Empathy, MC: Motivation/Curiosity, S: Skill, *e*: error covariance)

The EFA results suggested to keep as many of the original variables as possible (Table 4), which was a four-factor model with 24 items, named J-CCCHP24 (Fig. 3); removing three variables (KA 9, 25, and 30) and transferring three variables to other factors (MC 10 and 58 to S and KA 11 to A). The goodness-of-fit indices of J-CCCHP24 were similar to J-CCCHP22, but the Q-Q plots of J-CCCHP24 had fewer outliers from the straight diagonal line (Fig. 4). The Cronbach’s alpha of J-CCCHP24 was 0.85 and all subscales’ Cronbach’s alpha achieved above 0.60 (Table 2). Furthermore, the modification indices suggested adding four new correlation paths between error covariances (MC 38*e*–MC 64*e*, MC 64*e*–EE 48*e*, MC 58*e*–S 51*e*, and MC 12*e*–S 53*e*) to improve the goodness-of-fit indices (Fig. 5). As a result, J-CCCHP24 with error covariances indicated a better and more stable fit for the study data because of the goodness-of-fit indices: CFI = 0.92, TLI = 0.91, RMSEA = 0.05, SRMR = 0.06, and AIC = 16,559.6. All of these modifications seemed reasonable and well explained when read in the context of each item. For example, MC 10 (“By communicating, I can learn about different cultures”) seemed to be observing skill of cross-cultural communication instead of motivation/curiosity. MC 38*e*–64*e* may be both influenced by the personal characteristic of “proactive” as bias. Since nurses are interested in caring for foreign patients (MC 64), they desire to take advantage of training (MC 38).

**Table 4:** Exploratory factor analysis of the J-CCCHP24

		EFA (principal factor method and promax rotation)					CFA (ML)	
		MC	S	EE	A	Commonality	Correlation coefficient	Variance of error variables
MC	MC1	.80	-.09	-.09	.12	0.57	.74	0.45
	MC12	.58	.21	-.14	.15	0.55	.75	0.43
	MC17	.73	.02	-.15	.02	0.50	.70	0.51
	MC29	.58	.01	.02	-.02	0.35	.57	0.67
	MC38	.61	.04	.15	-.13	0.44	.59	0.65
	MC42	.78	.02	-.12	.16	0.65	.83	0.31
	MC64	.56	-.08	.47	-.23	0.53	.50	0.75
A	A8^a^	-.03	.06	.00	.46	0.23	.44	0.80
	A21^a^	-.15	-.00	.09	.42	0.20	.36	0.87
	A43^a^	.15	-.12	.17	.53	0.39	.67	0.56
	A60^a^	.17	.02	.05	.48	0.34	.60	0.64
	K11^a^	.08	-.03	-.08	.46	0.20	.41	0.83
EE	EE18^a^	-.10	-.01	.45	.08	0.21	.45	0.80
	EE26^a^	-.08	.03	.59	-.06	0.30	.49	0.76
	EE48^a^	.19	-.12	.65	-.01	0.45	.56	0.69
	EE55^a^	-.08	.01	.42	.22	0.29	.55	0.69
	EE63^a^	-.01	.12	.45	.08	0.21	.61	0.63
S	S5	-.04	.46	.10	.05	0.27	.50	0.75
	S44	.10	.48	.08	.05	0.38	.65	0.58
	S50	.16	.49	.08	.04	0.44	.71	0.49
	S51	.13	.64	-.12	-.00	0.47	.60	0.64
	S53	-.07	.72	.06	-.08	0.46	.60	0.64
	MC58	-.08	.79	-.14	.00	0.48	.56	0.69
	MC10	.20	.37	.07	-.09	0.27	.55	0.70
SS loadings		3.55	2.55	1.77	1.39			
Proportion var.		0.15	0.11	0.07	0.06			
Cumulative var.		0.15	0.25	0.33	0.39			

^a^﻿reversal items

### Known group method

Not J-CCCHP27 but J-CCCHP24 was used to carry out the known group method because its internal consistency was the most preferable. There was no significant difference in the mean scores of J-CCCHP24 between each group in gender, age, certification, and work experience, but it was significantly higher in participants who experienced more than 15 foreign patients compared to those who experienced 0 to 15 foreign patients (95.2 vs. 91.6) (Table 5).

**Table 5:** Comparison of J-CCCHP24 scores by characteristics

	*n*	%	Mean (SD)	95%CI	*P*-value for difference
J-CCCHP24	294	100	92.6 (10.8)	91.3–93.8	
Gender					0.08^a^
Female	243	82.7	93.1 (10.6)	91.7–94.4	
Male	51	17.3	90.2 (11.5)	86.9–93.4	
Age group					0.52^b^
20’s	71	24.1	94.1 (10.8)	91.5–96.6	
30’s	82	27.9	92.0 (10.2)	89.8–94.3	
40’s	86	29.3	92.6 (10.7)	90.3–94.9	
50’s and over	55	18.7	91.4 (11.6)	88.2–94.5	
Certification					0.35^a^
Nurse or Assistant nurse*	216	73.5	92.2 (10.9)	90.7–93.7	
Public Health nurse or Midwife	78	26.5	93.6 (10.5)	91.2–95.9	
Work experience					0.13^a^
0 to 5 years	67	22.8	94.3 (9.9)	91.9–96.7	
More than 5 years	227	77.2	92.1 (11.0)	90.6–93.5	
Number of foreign patients					0.01^a^
0 to 15	217	73.8	91.6 (10.5)	90.2–93.0	
More than 15	77	26.2	95.2 (11.3)	92.6–97.8	

^a^*t*-test

^b^ANOVA

^*^Assistant nurse, *n* = 3 (1%)

The results of the known group method showed significant differences in the mean scores of all four characteristics (Table 6). The mean scores for the four groups according to the level of subjective skill in foreign languages were significantly higher as the language level increased; not at all (87.3), greetings (92.1), beginner (95.8), and intermediate to advanced (101.1) (rho = 0.39, *p* < 0.001). The mean score when participants were divided into three groups based on intercultural interactions outside the workplace was higher in the group with regular cross-cultural interactions (98.1), the group with cross-cultural interactions in the past was in the middle (93.4), and the group with no experience with cultural interactions was the lowest (89.6) (rho = 0.28, *p* < 0.001).

**Table 6:** Known group method*

	*n*	%	Mean (SD)	95%CI	*P*-value for difference
Experience of being abroad					< 0.001^a^
Yes	212	72.1	93.9 (10.7)	94.4–95.3	
No	82	27.9	89.2 (10.4)	86.9–91.5	
Subjective skills in foreign languages					< 0.001^b^ rho = 0.39
Intermediate to advanced level (simple explanation to business level)	35	11.9	101.1 (10.2)	97.6–104.6	
Beginner level (simple sentence)	61	20.7	95.8 (10.5)	93.1–98.5	
Greetings	115	39.1	92.1 (9.6)	90.3–93.9	
Not at all	83	28.2	87.3 (9.7)	85.2–89.4	
Cross-cultural nursing care training					< 0.001^a^
Yes	48	16.3	97.3 (10.8)	94.2–100.4	
No	246	83.7	91.6 (10.6)	90.3–93.0	
Intercultural interactions outside the workplace					< 0.001^b^ rho = 0.28
Yes	53	18.0	98.1 (10.7)	95.2–101.1	
Yes, I had in the past but not recently	111	37.8	93.4 (10.2)	91.5–95.3	
Not at all	130	44.2	89.6 (10.4)	87.2–91.4	

^a^*t*-test

^b^Spearman’s rank correlation coefficient

^*^J-CCCHP24 was used

## Discussion

This is the first study of the Japanese version of the CCCHP to measure the cross-cultural competence of nurses in Okinawa. While utilizing as many existing variables as possible, 24 items with a four-factor model named J-CCCHP24 improved the goodness-of-fit and internal consistency for our data, thereby strengthening the factor structure validity and reliability. According to the results of the known group method, the J-CCCHP24 seemed to represent the cross-cultural competence of the respondents. Consequently, the factors that could improve cross-cultural competence among healthcare workers include: (1) learning about intercultural care, (2) improving communication skills with foreign patients, and (3) striving to have intercultural interactions outside the workplace.

There are a few possible reasons the KA subscale did not correlate with the data. First, the combination of two components in one subscale may have led to lower internal consistency (alpha coefficient for KA of Germany = 0.54, Finland = 0.28, and Japan = 0.31) [8, 12]. While three items cover general knowledge about health and cultural diversity (KA 9, 25, and 30), only one question asks about the self-awareness of the influence of one’s own culture (KA 11). Second, differences in culture, social norms, and policies in the medical field of participants may have caused two items (KA 9 and 25) to not be correlated with KA (*p* > 0.05). In particular, KA 9 (“The opportunities to receive healthcare services differ even if compared within the foreign population”) might have been difficult for our participants to envision, because the universal health insurance system has been well established in Japan. Since having the universal healthcare policy is also mandatory for most foreign residents, approximately 95% of foreign residents have been covered [25]. Therefore, the participants in this study may have believed that medical services are equally provided to all residents in Japan, though the quality of services may not be equitable due to language barriers, prejudice, and so on. On the contrary, a question such as K 11 is a very important item for Japanese healthcare professionals to reflect on their cultural awareness, because the risk of imposing care is increased if they are not aware that their own culture can be the basis for prejudice and misperception [1–5]. Adjusting the questions appropriately to the culture and the regulations of the local health system may be essential, in addition to increasing the observational variables of general cultural knowledge, to improve the reliability of KA. Indeed, the KA subscale could be the most difficult part to standardize as a universal cross-cultural scale, hence, testing a greater number of items regarding cultural knowledge and awareness may be required as suggested by Bernhard et al. [8].

The sample of our study was confined to one of the 47 prefectures in Japan. Nurses are regulated by the Act on Public Health Nurses, Midwives, and Nurses to ensure the quality of nursing service [26]. Nurses, public health nurses and midwives are licensed after passing the national examination upon three or more years of standardized training, whereas assistant nurses are licensed after passing the prefectural examination upon a minimum of two years of standardized training. Once qualified, all types of nurses can work anywhere in Japan. In 2020, the total number of active nurses was 1.66 million with the ratio of nurses, public health nurses, midwives, and assistant nurses at 77%, 4%, 2%, and 17% respectively, whereas 0.02 million with the ratio at 78%, 4%, 2%, and 16% in Okinawa [27]. In this study, 26% were public health nurses and midwives combined and only one percent were assistant nurses, thus, our sample is unlikely to be representative of generalities. The educational backgrounds of our participants were unknown, but they may have had more opportunity to learn transcultural nursing than the general population because transcultural nursing has been recognized as a fundamental subject in nursing programs at the university level since 2014 [28]. However, only 16% of our participants answered that they had training in cross-cultural nursing, and there was no significance between age groups in our data. This was a noticeable difference from the previous studies in Europe where a majority of participants answered that they had been trained on cross-cultural care [8, 12]. Our study indicates a situation where people are interested in intercultural care but lack the opportunity to receive training to improve their cross-cultural nursing skills, or the training did not sufficiently emphasize the importance of cross-cultural care due to a lack of expertise on the part of the trainers [28].

According to the results of the known group method, as more opportunities for cross-cultural exchange and care arise, there will be less resistance to cross-cultural care and higher cross-cultural competence. Those who had experience of caring for more than 15 foreign patients and those who had continuous intercultural interactions outside the workplace had higher CCCHP scores. Leininger stated that improving cross-cultural competence should be an ongoing process [2, 4]. 37% of our participants expressed resistance to taking care of foreign patients by agreeing with EE 48 (“I prefer treating patients from the same cultural background”), but if healthcare workers are exposed to patients with different cultures, it may slowly but surely change their cross-cultural competence. In previous CCCHP-based studies, more than 90% of participants had the same nationality as the study site (German = 91.4%, Finnish = 90.8%, and Japanese = 99.7%). In this study, 44% of the participants noted that they had never had cross-cultural interactions outside the workplace, indicating that, for some people, work is the only place where they can experience different cultures.

Our cross-cultural experience is helpful to provide better quality of care to foreign patients, however, many healthcare professionals may not have chosen the profession because they are interested in cross-cultural exchange [29]. If they do not proactively seek cross-cultural experiences on their own, providing them with opportunities to reflect and learn cross-cultural skills and cultural sensitivity would help. In this study, 79% of the participants agreed with MC 38 (“I want to use the training and instruction”), which is 5% higher than that in Kamibayashi et al.’s study in Tokyo (72%) [30]. This may suggest that our participants reflected on their cultural care skills in the process of answering the questions and found that they hope to learn more about cross-cultural competence. It is also known that nurses prefer to receive cross-cultural training as on-the-job training during their working hours [30]. Therefore, it is helpful to understand the learning needs of each group and organization and develop the most suitable training to improve cross-cultural competence at the workplace. The modified J-CCCHP (i.e., J-CCCHP24) may work as a scale to understand the learning needs and motivators of individuals and organizations with regard to transcultural care training.

The MC and S scores in the abovementioned CCCHP-based studies were high among the participants in all three countries. This suggests that the high level of cross-cultural competence may only be a rough estimation. The reasons our participants had higher MC and S scores could be their work environment and the “*chanpuru culture*” (mixed culture) of Okinawans. Okinawa Prefecture was the only region invaded by the U.S. military after World War II. Since then, 40,000–50,000 soldiers and their families have remained stationed in the prefecture, and also, the tourism industry has developed well since the war [31, 32]. There are designated medical facilities for the U.S. military, but some patients visit hospitals in Okinawa for specialized medical care and for convenience. In this study, 96% of the participants had experience caring for foreign patients and 72% had traveled overseas, which is higher than the national average, based on Noji’s study (*n* = 7494); 70% of the nursing staff had experienced caring for foreign patients and 29% had been abroad [10]. As a result, our participants could have higher MC and S scores compared to nurses in other prefectures.

### Limitations

One of the great strengths of the CCCHP is that this scale can be shared by a variety of medical professions. However, the sample of the present study was confined to nurses for feasibility reasons. Further studies on the modified J-CCCHP are necessary to test it with multidisciplinary teams in organizations or nationwide, including physicians, pharmacists, laboratory technicians, and therapists. Moreover, repeating validation of the J-CCCHP with nurses in a different prefectural setting may be beneficial. This is because our sample is not necessarily representative of Japanese nurses.

Not all of the subscales had a Cronbach’s alpha coefficient of more than 0.80, which means that the internal consistency was not high but fair. If some variables are appropriate to Japanese healthcare professionals, especially in terms of culture and experience, it may improve the content validity and the Cronbach’s alpha coefficient. In addition, other reliability tests should be conducted, such as test/re-test.

To translate the scale as a cross-cultural study, it was recommended to use more than one translation technique to ensure the equivalence, but also necessary to examine the meanings and connotations of words in the adapted language [33]. We did not invite Japanese experts to discuss the content of the questionnaire because CCCHP was not a new instrument, however considering how complicated it is to develop a universal cross-cultural instrument, it would be beneficial to confirm the content validation with experts in order to recognize the cross-cultural complexities.

## Conclusion

This study introduced the CCCHP in the context of Japanese nurses in the prefecture of Okinawa and found that the original five-factor model was not a good fit to the data. The best fit was found to be the four-factor model J-CCCHP24 in which the measurement equation model was modified according to the results of EFA and modification index. The known group method showed a significant difference in the mean scores of groups divided by the characteristics as anticipated, suggesting that it can serve as a tool to measure the cross-cultural competence of Japanese nurses. However, since the data of this study were limited to only nursing staff in Okinawa Prefecture, it is necessary to further evaluate the reliability and practicality by conducting research in health care professions other than nursing and in other parts of Japan. This study can be said to be a blueprint for developing a tool to measure cross-cultural competence among Japanese healthcare professionals.

### Supplementary Information

**Supplementary Material 1. **

**Supplementary Material 2. **

## Data Availability

The datasets used in this current study are available from the corresponding author on reasonable request.

## Abbreviations

A: Attitude
AIC: Akaike’s information criteria
CCCHS: The Caffrey Cultural Competence in Healthcare Scale
CCCHP: Cross-Cultural Competence instrument for Healthcare Professionals
CFA: Confirmatory factor analysis
CFI: Comparative fit index
EE: Emotion/Empathy
EFA: Exploratory factor analysis
J-CCCHP: Japanese version of Cross-Cultural Competence instrument for Healthcare Professionals, 5 factors and 27 items
J-CCCHP22: Modified Japanese version of Cross-Cultural Competence instrument for Healthcare Professionals, 4 factors and 22 items
J-CCCHP24: Modified Japanese version of Cross-Cultural Competence instrument for Healthcare Professionals, 4 factors and 24 items
JMIP: Japan Medical Service Accreditation for International Patients
KA: Knowledge/Awareness
KMO: Kaiser-Mayer-Olkin measure of sampling adequacy
MC: Motivation/Curiosity
Q-Q plot: Quantile–Quantile plot
RMSEA: Root mean square error of approximation
S: Skill
SD: Standard deviation
sk: Skewness and kurtosis
SRMR: Standardized root mean squared residual
TLI: Tucker-Lewis index

## References

[1] Teraoka M, Muranaka Y (2017). Aspects of cross-cultural experience perceived by foreigners living in Japan when using its healthcare services. J Jpn Acad Nurs Sci..

[2] Leininger MM. What is transcultural nursing and culturally competent care? J Transcult Nurs. 1999;10(1):9. 10.1177/104365969901000105. 10.1177/104365969901000105 10476143

[3] Ono S, Yamamoto Y. Cultural competence in nursing: a literature review. Kawasaki Med Welf J. 2011;20(2):507–12. 10.15112/00013174.

[4] McFarland MR, Wehbe-Alamah HB. Leininger’s Transcultural Nursing: concepts, theories, research & practice. 4th edition. McGraw-Hill Education; 2018.

[5] Tseng WS, Streltzer J. Cultural Competence in Health Care. New York: Springer; 2008.

[6] Nomura Research Institute. Report on the projects to promote the establishment of medical technology services: survey on the promotion of acceptance of foreign patients by Japanese medical institutions. Mar 2016. Retrieved from: https://www.meti.go.jp/policy/mono_info_service/healthcare/iryou/downloadfiles/pdf/27fy_inbound_NRI.pdf. Accessed 30 Dec 2023.

[7] Ministry of Health, Labour and Welfare. Medical facilities current status survey: approximate number at the end of September 2022. Retrieved from: https://www.mhlw.go.jp/toukei/saikin/hw/iryosd/m22/dl/is2209_01.pdf. Accessed 30 Dec 2023.

[8] Bernhard G, Knibbe RA, von Wolff A, Dingoyan D, Schulz H, Mösko M. Development and psychometric evaluation of an instrument to assess cross-cultural competence of healthcare professionals (CCCHP). PLoS ONE. 2015;10(12):e0144049. 10.1371/journal.pone.0144049. 10.1371/journal.pone.0144049 PMC4671537 26641876

[9] Sugiura K. Survey-based analysis of cultural competence in nursing and its predictors: comparison between nurses who were Japan Overseas Cooperation Volunteers and nurses working at municipal hospitals. J Jpn Acad Nurs Sci. 2003;23(3):22–36. 10.5630/jans1981.23.3_22.

[10] Noji A, Mochizuki Y, Nosaki A, Glaser D, Gonzales L, Mizobe A, Kanda K. Evaluating cultural competence among Japanese clinical nurses: analyses of a translated scale. Int J Nurs Pract. 2017;23(Suppl 1):e125551. 10.1111/ijn.12551. 10.1111/ijn.12551 28635065

[11] Caffery RA, Neander W, Markle D, Stewart B. Improving the cultural competence of nursing students: results of integrating cultural content in the curriculum and an international immersion experience. J Nurs Educ. 2005;44(5):234–40. 10.3928/01484834-20050501-06. 10.3928/01484834-20050501-06 15916027

[12] Hietapakka L, Elovainio M, Wesolowska K, Aalto A, Kaihlanen A, Subervi T, Heponiemi T (2019). Testing the psychometric properties of the Finnish version of the cross-cultural competence instrument of healthcare professionals (CCCHP). BMC Health Serv Res..

[13] Kyriazos TA (2018). Applied psychometrics: sample size and sample power considerations in factor analysis (EFA, CFA) and SEM in general. Psychology..

[14] Okinawa Prefecture. About medical institutions available for foreign language(s). Retrieved from: https://www.pref.okinawa.lg.jp/_res/projects/default_project/_page_/001/006/367/gaikokujinkannjyaukeirekanou.pdf. Accessed 30 Dec 2023.

[15] Inoue T, Kurata Y. The intellectuals who defend the foreign workers policy neglecting the existence of migrants (1) multicultural co-existence society. Hitotsub Bull Soc Sci. 2020;12:27–36. 10.15057/31041.

[16] Onodera N. Research note: different responses to “Very” and “Quite” - an examination of alternative wording in international comparative surveys. The NHK monthly report on broadcast research. 2002-Jan;52(1):62–75. Retrieved from: https://www.nhk.or.jp/bunken/summary/yoron/method/pdf/040901.pdf. Accessed 30 Dec 2023.

[17] Williams B, Onsman A, Brown T (2010). Exploratory factor analysis: A five-step guide for novices. Australas J Paramedicine..

[18] Barton B, Peat J. Descriptive statistics*. *In Medical Statistics: a guide to SPSS, data analysis and critical appraisal. 2nd edition. Edited by Barton B, Peat J. Oxford: John Wiley & Blackwell; 2014. p. 24–51.

[19] Yoshida T, Ishihara H, Haebara T (2012). Construction, use, and validation of psychological scales. Ann Rep Educ Psychol Jpn..

[20] Cummings SR, Kohn MA, Hulley SB. Designing questionnaires, interviews, and online surveys. In Designing Clinical Research. 4th edition. Edited by Hully SB, Cummings SR, Browner WS, Grady DG, Newman TB. Philadelphia: Lippincott Williams & Wilkins, a Wolters Kluwer business; 2013. p. 223–30.

[21] Hu L, Bentler PM (1998). Fit indices in covariance structure modeling: sensitivity to under parameterized model misspecification. Psychological Method..

[22] Hoshino T, Okada K, Maeda T (2005). Fit indices and model modification in structural education modeling: a review and new findings. Jpn J Behaviormetrics..

[23] Kawahashi I, Iwama N, Suzuki M (2018). Introduction to Multivariate Analysis with R: practice and theory of data analysis.

[24] Kano Y (2002). Rejoinder: use of error covariances and the role of specific factors. Jpn J Behaviormetrics.

[25] Survey Research Center Co., LTD. The second comprehensive survey of foreign residents: insurance and pension for foreign residents. 26 May 2022. Retrieved from: https://www.surece.co.jp/wp_surece/wp-content/uploads/2022/05/2022052610.pdf. Accessed 30 Dec 2023.

[26] Japanese Law Translation. Act on public health nurses, midwives, and nurses (Act No. 203 of 1948). Act No. 78 of 2009. Retrieved from: https://www.japaneselawtranslation.go.jp/en/laws/view/3993. Accessed 30 Dec 2023.

[27] Ministry of Health, Labour and Welfare. Overview of the year 2020 health administration reports (for employed medical personnels). Retrieved from: https://www.mhlw.go.jp/toukei/saikin/hw/eisei/20/dl/gaikyo.pdf. Accessed 26 Dec 2023.

[28] Ono N. Cultural competence in medical settings. Juntendo J Global Studies. 2016;1:70–79. https://www.juntendo.ac.jp/academics/faculty/ila/assets/1-20160325hp.pdf#page=74. Accessed 30 Dec 2023.

[29] Gasiorek J, Van de Poel K (2018). Language-specific skills in intercultural healthcare communication: comparing perceived preparedness and skills in nurses’ first and second languages. Nurse Educ Today..

[30] Kamibayashi C, Kondo A, Koizumi M, Futami A. Needs and motivation of hospital nurses regarding training on how to care for foreign patients in Japan. J Int Health. 2020;35(1):27–38. 10.11197/jaih.35.27.

[31] Yamamoto K. Medical care for foreigners in Okinawa prefecture: current status and issues in treating tourists and long-term residents. J Okinawa Med Assoc. 2019;55(4):18–26.

[32] JICA Okinawa. Survey report on the current situation and issues of foreign human resources and multicultural society in Okinawa. April 2021. Retrieved from: https://www.jica.go.jp/okinawa/topics/2021/ku57pq00000mi1o4-att/ku57pq00000mi1ow.pdf. Accessed 26 Dec 2023.

[33] Cha ES, Kim KH, Erlen JA (2007). Translation of scales in cross-cultural research: issues and techniques. J of Adv Nurs..

