# Supplementary material for: Evaluating the cross-cultural competence instrument for healthcare professionals (CCCHP) among nurses in Okinawa, Japan
Source: BMC Health Serv Res. 2024 Mar 23;24:369. doi: 10.1186/s12913-024-10814-6 (PMC10960992; doi:10.1186/s12913-024-10814-6)
Supplement: Supplementary file 1 — Supplementary Material 1. [file 12913_2024_10814_MOESM1_ESM.docx]

**Supplementary file 1: Japanese-CCCHP**

|  | items | English version | Japanese version | |
| --- | --- | --- | --- | --- |
| MC | MC 1 | I consider working in a cross-cultural team an enrichment | 外国人スタッフと一緒に働くことは豊かさにつながると思う。 | |
|  | MC 10 | By communicating with patients with a migration background I can learn about different cultural orientations. | 外国人患者さんと話してみると文化の違いによって考え方が異なることがわかる。 | |
|  | MC 12 | Cultural diversity is also an enrichment. | 文化の多様性も一つの豊かさだと思う。 | |
|  | MC 17 | I enjoy talking to people who have migrated to [country concerned] about their experiences here. | 在留外国人から日本での体験談を聞くと面白い。 | |
|  | MC 29 | The interaction with people from other cultural backgrounds helps me reflect upon my own cultural background. | 私と異なる文化背景の人との交流は、自分の文化背景を振り返ってみることを助ける。 | |
|  | MC 38 | I would like to make use of training, advising and educational offers, in order to improve my understanding of patients with a migration background. | もっと外国人患者さんのことを理解できるように、研修・指導・講習などを活用したい。 | |
|  | MC 42 | I consider it an enrichment to have friendships with people from different cultural backgrounds. | 私と異なる文化背景の人と友情を育むことは豊かさにつながると思う。 | |
|  | MC 58 | It is important for me to treat patients according to their cultural needs and individual values, | 患者さんの文化的要求や価値観に合った治療やケアを提供することが重要である。 | |
|  | MC 64 | I find it exciting to treat patients with a migration background. | 外国人患者さんの治療やケアに従事することはワクワクする。 | |
| A | A 8* | I find it an imposition, when people who migrated to [country concerned] a long time ago, cannot speak [language concerned] properly. | 長い間日本にいる在留外国人が日本語を正しく話せないのはおかしいと思う。 | |
|  | A 21* | I have the impression that migrants often assume discrimination, when in fact general rules are simply being enforced. | 実際には一般的なルールが適応されているだけなのに、在留外国人は「差別」と感じていることが多い。 | |
|  | A 43* | People who migrate to [country concerned] should adapt to society, not the other way around. | 日本に移り住む人が日本社会に適応すべきであり、その逆ではないと思う。 | |
|  | A 60* | Institutions and the public pay too much attention to the special wishes of migrants. | 日本の医療機関や行政サービスは在留外国人の特別な要望を考慮しすぎている。 | |
| KA | KA 9* | Within the migrant population, there are hardly any differences in terms of health opportunities and disease risks. | 在留外国人において保健医療サービスを利用する機会や病気にかかる機会は、皆同じだと思う。 | |
|  | KA 11* | My professional perception, assessment, and behavior remain untouched by my cultural imprinting. | 私の中に潜在的に刷り込まれている文化が、医療従事者としての自覚・アセスメント能力・行動を変えることはない。 | |
|  | KA 25 | The migration experience is a critical life event and can be accompanied by psychosocial stress and health burden. | 外国に移り住むことは重大なライフイベントであり、心理社会的ストレスや健康上の問題を伴うことがある。 | |
|  | KA 30* | The disease concepts of patients with a migration background are not relevant for treatment success. | 外国人患者さんの疾病理解と治療の成功は関係がない。 | |
| *Indicates reversal items on a 5-point Likert scale, where 1 = 1 point for reversal items and 1 = 5 points for normal items | | | |  |

|  | items | English version | | Japanese version | | |  |
| --- | --- | --- | --- | --- | --- | --- | --- |
| EE | EE 18* | I often find it difficult to relate to the elaborations of my patients, when their socio-cultural background is quite different from my own. | | 私と患者さんの社会文化的背景がまったく異なる場合、患者さんからどんなに詳しい説明を聞いてもその人の立場に立って理解することは難しい。 | | |  |
|  | E26* | I find it difficult to speak slowly in lay language with people who struggle to understand my instructions. | | 私の指示や説明が理解できずに困っている患者さんに易しい言葉だけでゆっくり話すのは難しい。 | | |  |
|  | EE 48* | | I prefer treating patients from my own cultural background, than those who seem foreign to me. | | どちらかと言えば、明らかに異なる文化背景の患者さんより自分と同じ文化背景を持つ患者さんを担当したい。 | | |
|  | EE 55* | | In my professional interaction with patients with a migration background, I often feel unsure, angry and frustrated. | | 医療従事者として外国人患者さんと関わると、自分に自信がなくなったり怒りや苛立ちを感じたりすることが多い。 | | |
|  | EE 63* | | I get impatient when I cannot make myself understood with a migration background. | | 外国人患者さんに自分の言うことを理解してもらえないとイライラする。 | | |
| S | S 5 | | In order to achieve the agreed treatment goal, I ask patient with a migration background what they need in terms of support. | | 外国人患者さんが同意した治療目標を達成するためにどのような支援が必要か、患者さん本人に確かめる。 | | |
|  | S 44 | | With patients who do not understand [language concerned] very well, I take more time to discuss their expectations and fears. | | 日本語を十分に理解できない患者さんには、いつもより時間をかけてそれぞれの期待や不安を話し合うようにする。 | | |
|  | S 50 | | With patients who do not understand [language concerned] very well, I take more time to explain the treatment options to them. | | 日本語を十分に理解できない患者さんには、いつもより時間をかけて治療の選択肢などを説明する。 | | |
|  | S 51 | | Culturally specific factors of people (e.g. values, behaviour norms, beliefs) influence their understanding of disease significantly, and should therefore be assessed and taken into consideration by healthcare professionals. | | 一人ひとりが持っている文化的な特徴（例えば価値観、行動規範、信念）は、疾病理解に大きな影響を与えるので、医療従事者は十分に評価して考慮するべきである。 | | |
|  | S 53 | | I consider the values of patients in relation to family, religion, etc., if they seem relevant for the treatment. | | もし治療に影響しそうなら、家族や宗教など、患者さんが大切にしていることも考慮する。 | | |
| SD | SD 1 | | I do not differentiate between patients and treat all equally, though it is sometimes difficult to communicate. | | コミュニケーションが取りづらいと感じる時があっても、患者さんを区別せず平等に接している。 | | |
|  | SD 2 | | I always remain friendly and courteous with people from a different cultural background, even when I am stressed out. | | ストレスを感じている時であったとしても、私は異なる文化背景の人にはいつも友好的で礼儀正しくしている。 | | |
|  | SD 3 | | In a conversation I always listen attentively and let individuals with a migration background finish their sentences. | | 患者さんと会話する時は傾聴に心がけており、相手が在留外国人ならその人が最後の一言まで言えるようにする。 | | |
|  | SD 4 | | During arguments with people from a different cultural background, I always remain factual and objective. | | 私と異なる文化背景の人と言い争いになったら、事実と客観的な視点から外れないようにする。 | | |
|  | SD 5 | | I never hesitate to help someone with a different cultural background in case of emergency. | | 生命の危機に直面している時は、私と異なる文化背景の人でもためらわずに助ける。 | | |
| *Indicates reversal items on a 5-point Likert scale, where 1 = 1 point for reversal items and 1 = 5 points for normal items | | | | | |  |  |
