# Supplementary material for: Evaluating the cross-cultural competence instrument for healthcare professionals (CCCHP) among nurses in Okinawa, Japan
Source: BMC Health Serv Res. 2024 Mar 23;24:369. doi: 10.1186/s12913-024-10814-6 (PMC10960992; doi:10.1186/s12913-024-10814-6)
Supplement: Supplementary file 2 — Supplementary Material 2. [file 12913_2024_10814_MOESM2_ESM.docx]

**Supplementary file 2: Answer options of CCCHP**

| Points* | 5 | 4 | 3 | 2 | 1 | 0 |
| --- | --- | --- | --- | --- | --- | --- |
| German version | Completely agree | Mostly  agree | Neither agree nor disagree | Mostly  disagree | Completely disagree | No answer possible |
| Finnish version | Fully  agree | Mostly agree | Neither agree nor disagree | Mostly disagree | Fully disagree |  |
| Japanese version | Agree | Mostly agree | Neither agree nor disagree | Mostly disagree | Disagree |  |

*: Reversal points were assigned for 12 reversal items.
